# One-year follow-up of Atraumatic Restorative Treatment(ART) for dental caries in children undergoing oncohematological treatment: a pragmatic trial

**DOI:** 10.1186/s12903-015-0110-y

**Published:** 2015-10-16

**Authors:** Cíntia Ferreira Gonçalves, Mariana Vargas Lindemaier e Silva, Luciane Rezende Costa, Orlando Ayrton de Toledo

**Affiliations:** Health Sciences Graduate Program, University of Brasília, Brasília, Federal District Brazil; Dentistry Graduate Program, São Leopoldo MandicUniversity, Campinas, São Paulo Brazil; Division of Pediatric Dentistry, Faculdade de Odontologia, Universidade Federal de Goiás, Goiânia, Goiás Brazil; Avenida Teotônio Segurado, Cj. 01, Lt. 01, Sl. 508, Plano Diretor Sul, Palmas, TO CEP: 77061-002 Brazil

**Keywords:** Dental care for children, Leukemia, Lymphoma, Antineoplastic drugs combination, Dental caries, Dental atraumatic restorative treatment

## Abstract

**Background:**

The oral condition in children undergoing oncohematological treatment can have a negative impact on the course of disease. Little is known about survival of tooth restorations in these patients. The aim of this study was to evaluate the longevity of restorations and sealants performed by Atraumatic Restoration Treatment (ART) in patients undergoing oncohematological treatment.

**Methods:**

ART single surface restorations and sealants were performed in the experimental group (E), which comprised children (2–13 years old) undergoing oncohematological treatment, and in the control group (C), in which patients did not undergo such treatment. The same examiner evaluated the ART at 1, 3, 6 and 12 months after preparation, using the same criteria for restorations and sealants. ART was successful if the sealant or restoration did not need a repair in the follow-up assessment. Descriptive, bivariate and Cox’s proportional hazard analyses were performed at a significance level of 5 %.

**Results:**

The two groups, one including 24 children (E) and the other 14 children (C), received 101 and 52 ART procedures, respectively. The success rates were 95.0 % (E) and 100 % (C) at 1 month (*P* = 0.233); 81.2 % (E) and 92.3 % (C) at 3 months (*P* = 0.009); 72.2 % (E) and 80.8 % (C) at 6 months (*P* = 0.050) and 48.5 % (E) and 73.1 % (C) at 12 months (*P* = 0.001). The final Cox’s regression model for occurrence of ART failure needing repair did not show differences between groups (E: OR = 1.6, 95 % CI 0.8–2.9); primary teeth had a shorter survival than permanent teeth (OR = 2.1, 95 % CI 1.2–3.7).

**Conclusions:**

Oncohematological treatment did not interfere with the longevity of ART restorations and sealants, which suggests the potential use of this technique in children undergoing chemotherapy.

**Trial registration:**

*Brazilian Clinical Trials Registry (REBEC)* RBR-2c3c52. Registered 5 June 2014. http://www.ensaiosclinicos.gov.br/rg/RBR-2c3c52/

**Electronic supplementary material:**

The online version of this article (doi:10.1186/s12903-015-0110-y) contains supplementary material, which is available to authorized users.

## Background

The oral condition of children and adolescents undergoing oncohematological treatment requires frequent monitoring during the course of the antineoplastic therapy [1, 2]. These patients usually present highly compromised immunity, including the deterioration of the oral health status, gingival status and increased experience of dental caries [3–5]. Advanced dental caries in these children may represent significant morbidity and a source of general infection withpotential risk of mortality [6]. Moreover, dental caries is one of the most observed late effects of oncohematological treatment in survivors of childhood cancer [7, 8]. Therefore, it is important for children’s overall health that a specialized dentist manages their oral health condition from the pre-cancer therapy phase on ward to avoid the negative impacts of possible oral infections [9].

Another important consideration is that the salivary flow rate, salivary pH and total salivary antioxidant levels are lower in leukemic children compared with controls [3]. Thus, to achieve successful systemic treatment, it is necessary to seal open carious cavities and active white spots because oncohematological treatment contributes to the compromise of salivary defense mechanisms, increasing the possibility of local infections [10]. Moreover, salivary sialic acid levels have potent activity in the dental caries process, and patients with acute lymphocytic leukemia (ALL) present salivary sialic acid levels higher than those of controls [4]. Another important factor is the presence of excess nitric oxide in the saliva, which may be associated with higher rates of mucosal lesions and periodontal disease in these children [11].

Atraumatic Restorative Treatment (ART) could benefit children before the initiation of cancer treatmentby allowing a more conservative approach to dental caries management compared with conventional restorative dental treatment. The ART was first introduced in underserved populations, where the access and availability of dental equipment for conventional techniques are limited. This technique was based on scientific findings about partial caries removal with minimally invasive dentistry: the removal of demineralized dentin using hand tools and without the need for local anesthesia; disinfection of the cavity; and tooth restoration. In the ART approach, the repair of cavities and sealing of pits and fissures are performed by digital pressure with high-viscosity glass ionomer and the protection afforded by this material [12]. Currently, ART is defined as a minimally invasive approach to prevent dental caries and stop its future progression [12]. Thus, it has become possible to apply more conservative dentistry, preserving more dental tissues than in the past [13].

The number of publications reporting on the survival of atraumatic restorative treatment (ART) has increased considerably. Nonetheless, we found only one study involving ART in people with special needs (most of them with cerebral palsy and autism), which compared the survival of ART restorations with composite resin restorations in, and as a result, ART restorations had better survival rates [14]. Current research shows that the longevity of ART is similar to conventional techniques in patients without special needs [13]. To the best of our knowledge, no study has compared ART with conventional dental restorations in children undergoing oncohematological treatment. Moreover, the ART has great benefits as an effective restorative technique, since it preserves more dental tissue than conventional dental treatment. In addition, it would avoid the use of rotary equipment and dental anesthesia, which are known to contribute to anxiety during dental treatment [15]; and it is important remember that these patients constantly undergo hospital manipulations for chemotherapy, which are potentially related to increased anxiety and fear [16]. Then, the ART could be recommended for people who go through stressful procedures in hospitals because it would cause less pain and anxiety [15].

Considering that children receiving oncohematological treatment present salivary changes that would make them more prone to caries and consequently unsuccessful restorations [17], our hypothesis was: these children would benefit from the ART for a period of time but would have more ART failure in the long-term. The aim of this study was to evaluate the longevity of restorations and sealants using the ART technique in children undergoing oncohematological treatment compared with healthy children.

## Methods

### Study design, setting and ethical aspects

This pragmatic prospective controlled clinical trial was conducted in the city of Palmas, capital of the State of Tocantins, North of Brazil. Tocantins is a state of 1,496,880 people, with one public hospital that provides oncohematological treatment for the underserved population. Children included in the experimental group came from the Palmas General Public Hospital (HGPP).

Ethical approval was obtained from the Federal University of Tocantins Research Ethical Committee (protocol #011/2012) and register number in REBEC, RBR-2c3c52. Informed consent was granted by patients’ parents after they were informed about the study’s aim, procedures, risks and benefits.

### Participants and intervention

For the experimental group, all children aged between 2 and 13 years old who were undergoing oncohematological treatment at the HGPP during 2012 were assessed for eligibility. Children were included if they had presence of biofilm, active white spot and/or active cavities, despite the stage of the oncohematological treatment – initiation or remission. Those patients whose systemic involvement was too advanced to allow dental manipulation, such as those who were admitted to the intensive care unit, were excluded from the study. Immunosuppression alone was not an exclusion criterion.

The control group was selected among healthy children from the same age range who attended the pediatric dental clinic at the Brazilian Dental Association – Tocantins section (ABO-TO). The control group children were also at risk/activity of dental caries. None of the patients had any type of special need or were undergoing any type of oncohematological treatment.

Children from both groups participated in a basic oral health program, including dietary counseling and oral hygiene instruction, and the patients who required dental procedures other than those planned in the present study, such as the restoration of proximal cavities and extractions, were referred to the department of dental oncology in the HGPP or the ABO-TO clinic.

The participants were examined and treated by one dentist certified in pediatric dentistry and in dentistry for patients with special care needs (around 750 hundred hours each program). This dentist received didactic and practical training in ART performance during the specialization programs. The ART procedures were done and evaluated by the same operator.

In the first dental consultation, the dentist recorded the participants’ characteristics and the presence of dental caries, assessed as white spots, decayed, missing, and filled teeth (dmft for primary teeth or DMFT for permanent ones), according to the criteria of the WHO Oral Health Surveys Basic Methods [18]. The patients were seen on a stretcher in the outpatient oncology hospital room, simulating the conditions recommended by the original ART technique [12]. The teeth expected to exfoliate intwelve months did not receive ART.

After the child was positioned on the stretcher, the dentist performed the ART with a flashlight under cotton roll isolation, using hand instruments only (ART Kit, Henry Schein, Chicago, IL, USA) and a mouth prop. Children seen in the dental clinic were positioned in the dental chair. The sealant and restorative material was the high-viscosity glass ionomer Ketac Molar Easymix (3M ESPE, St. Paul, MN, USA), which was used according to the manufacturer’s instructions. Class I restorations and sealants were performed on the occlusal surfaces of molars and the palatal surface of some incisors, and Class V restorations were performed on the buccal surface of molars. In all cavities, softened infected carious tissue was removed from dentinal lesions in primary and permanent teeth based on tactile and optical criteria using hand instruments, according to the ART protocol [12]. In all procedures, the enamel or dentin surface was conditioned with the liquid component. The glass ionomer cement was hand-mixed according to the manufacturer’s instructions. The cavity and adjacent fissures were filled and held under finger pressure for 60 s. Excessive cement was removed with hand instruments. A layer of Vaseline was placed over the restoration or sealant to maintain the water balance in the glass ionomer cement. Then, the occlusion was checked with a carbon paper (Accu Film II, Parkel, Farmingdale, NY, USA).

### ART quality assessment

The quality of the ART sealants and restorations was carried out by the same examiner who performed the procedures, in the follow-up sessions scheduled for 1, 3, 6 and 12 months post-treatment using established ART restoration criteria by Roeleveld et al., [19] (Table 1), adapted to include dental sealants. Codes 00 and 10 were considered success, and the other codes, failure; the code 10 is related to a little failure that does not require repair, so it was categorized as success. This examiner reassessed the sealants and restorations in 10 % of the sample in an interval of 2 weeks, with a weighted Kappa coefficient of 0.707 for intra-examiner consistency.

**Table 1:** Criteria for follow-up evaluation of ART restorations and sealants (according to Roeleveld et al., [19])

Code	Evaluation characteristics
00	Restoration present, correct
10	Restoration present, slight marginal defect/wear of surface (<0.5 mm). No repair needed.
11	Restoration present, gross marginal defect/wear of surface (>0.5 mm). Repair needed.
12	Restoration present, underfilled (>0.5 mm). Repair needed.
13	Restoration present, overfilled (>0.5 mm). Repair needed.
20	Secondary caries, discoloration in depth, surface hard and intact, caries within dentin. Repair needed.
21	Secondary caries, surface defect, caries within dentin. Repair needed.
30	Restoration not present, bulk fracture, moving or partial lost. Repair needed.
40	Inflammation of the pulp; signs of dentogenic infection (abscesses, fistulae, pain complaints). Restoration might still be in situ. Extraction needed.
50	Tooth not present because of extraction
60	Tooth not present because of shedding
70	Tooth not present because of extraction or shedding
90	Patient not present

### Statistical analysis

Data were analyzed by one researcher using IBM SPSS 19.0 and checked by another member of the research team.

For initial analysis, data were analyzed according to the experimental group by calculating frequencies and measures of dispersion as appropriate. Then, Fisher’s Exact Test, Pearson’s Chi-Square and the Mann–Whitney U Test were used to compare the experimental and control groups regarding children’s demographics, caries experience and ART. Significance was set at *P* <0.05.

To analyze the failure of ART restorations and sealants in each return visit (follow-up session as described earlier) as a function of predictor variables, survival analysis was chosen instead of regression or ANOVA because it allowed for censored values such as cases lost to follow-up. The response variable was the time elapsed between the baseline consultation, when the restorations and sealants were applied, and the end of follow-up or the censorship of cases. The censored cases were those that had abandoned the follow-up after at least one ART assessment, or those who had lost all procedures. If follow-up data were completely missing for any subject (missing data), that case was left out.

Cox’s proportional hazard model evaluated explanatory variables for the restorations’ and sealants’ survival, including sex, age, and type of behavioral intervention. This model is a multiple regression analysis applied to survival analysis and is indicated when an estimate of the role of independent variables that act multiplicatively on risk is desired. The assumption was that individuals undergoing chemotherapy have different survival rates for restorations and sealants than those individuals who are not undergoing this type of treatment.

## Results

### Characteristics of participants

**Fig. 1:**
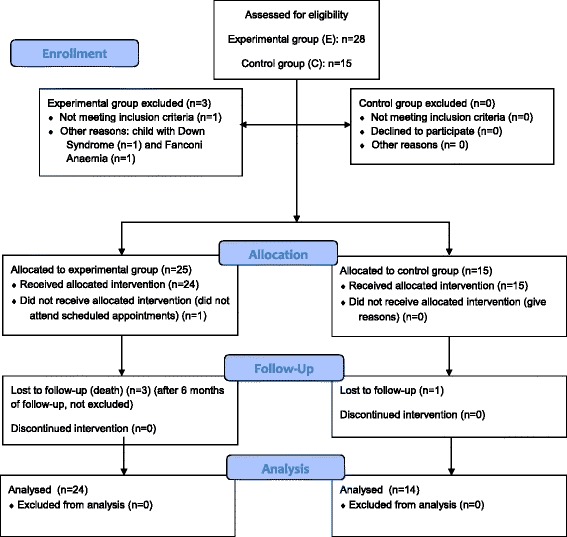
Flow diagram of participants in this quasi-experimental clinical trial

A total of 38 children were included in the study, 24 in the experimental group and 14 in the control group (Fig. 1). There were no differences between the experimental (oncohematological) and control (healthy) groups regarding children’s demographics, caries experience and number of ART procedures performed (Table 2). Among the total of 24 patients undergoing oncohematological treatment, the greatest incidence of underlying pathology was acute lymphoblastic leukemia (ALL), including its subdivisions, such as high risk, low risk, and intermediate risk. In addition, some patients in the experimental group were in treatment for some types of lymphoma. Among the protocol’s recommended treatments, the most frequent was the European Berlin-Frankfurt-Munster (BFM-90) group protocol, adopted by the Brazilian National Cancer Institute, which recommends short, intensive multidrug leukemia therapy. Half of the sample was subjected to radiotherapy treatment. None of the patients in the experimental group had undergone bone marrow transplantation by the time of the study.

**Table 2:** Participants’ characteristics

Variables	Experimental (*n* = 24)	Control (*n* = 14)	*P*
Children demographics			
Sex, n (%)			
Female	10 (41.7 %)	8 (57.1 %)	0.503^a^
Male	14 (58.3 %)	6 (42.9 %)	
Age, median (range)	7.0 (2.0–13.0)	7.5 (4.0–13.0)	0.893^b^
Caries experience			
DMFT, median (range)	1.0 (0–10.0)	2.0 (0–6.0)	0.622^b^
White spots, median (range)	2.0 (0–8.0)	3.5 (0–4.0)	0.235^b^
ART (single surface restorations and sealants)			
Number of procedures/child, median (range)	4.0 (2.0–14.0)	4.0 (2.0–8.0)	0.612^b^

^a^Fisher’s Exact Test; ^b^Mann-Whitney *U* Test

A total of 101 ART procedures were performed in the experimental group, and 52 were performed in the control group. Groups did not differ regarding the baseline characteristics of the ART (teeth ant type of procedure), but there were differences in a few follow-up periods (Table 3). All but one child had positive or definitely positive behavior [20]. Most ART procedures were performed in permanent teeth and were of the sealant type. In the follow-up period, teeth that needed ART repair were excluded from the subsequent analyses because they required new restorations. At the third and twelfth months of follow-up, groups did not differ in codes 00 and 20, but there were more Code 10 ART in the control group and Code 30 ART in the experimental group (*P* <0.05).

**Table 3:** Characteristics of the Atraumatic Restorative Treatment (ART) procedure

Variables	Experimental (*n* = 101 ART)	Control (*n* = 52 ART)	*P*
Teeth			1.000^*^
Primary molars	27 (26.7 %)	14 (26.9 %)	
Permanent	74 (73.3 %)	38 (73.1 %)	
Lateral incisors	4 (4.0 %)	0	
Premolars	7 (7.0 %)	0	
Molars	63 (62.3 %)	38 (73.1 %)	
Type of ART			0.297^†^
Sealant	88 (87.1 %)	42 (80.8 %)	
Restoration	13 (12.9 %)	10 (19.2 %)	
Tooth surface			0.114^†^
Occlusal	93 (92.1 %)	52 (100 %)	
Palatal	4 (4.0 %)	0	
Vestibular	4 (4.0 %)	0	
One-month follow-up			
Evaluation^‡^			0.233^†^
Code 00	89 (88.1 %)	47 (90.4 %)	
Code 10	7 (6.9 %)	5 (9.6 %)	
Code 30	5 (5.0 %)	0	
Type of failure			–
Partial loss, distal	4 (4.0 %)	1 (1.9 %)	
Partial loss, lingual	1 (1.0 %)	0	
Partial loss, mesial	2 (2.0 %)	4 (7.7 %)	
Total loss	5 (5.0 %)	0	
Three-month follow-up			
Evaluation^‡^			0.009^†^
Code 00	80 (79.2 %)	41 (78.8 %)	
Code 10	2 (2.0 %)	7 (13.5 %)	
Code 20	1 (1.0 %)	1 (1.9 %)	
Code 30	13 (12.9 %)	3 (5.8 %)	
Type of failure			
Partial loss, distal	2 (2.0 %)	3 (5.8 %)	
Partial loss, distal with dentin exposure	1 (1.0 %)	0	
Partial loss, mesial	0	3 (5.8 %)	
Total loss	13 (12.9 %)	3 (5.8 %)	
Tooth decay, distal	0	1 (1.9 %)	
Six-month follow-up			
Evaluation^‡^			0.050^†^
Code 00	67 (66.3 %)	35 (67.3 %)	
Code 10	6 (5.9 %)	7 (13.5 %)	
Code 20	0	2 (3.8 %)	
Code 30	5 (5.0 %)	4 (7.7 %)	
Child died	4 (4.0 %)	0	
Type of failure			
Partial loss, distal	3 (3.0 %)	3 (5.8 %)	
Partial loss, mesial	2 (2.0 %)	4 (7.7 %)	
Total loss	4 (4.0 %)	4 (7.7 %)	
Tooth decay, distal	0	1 (1.9 %)	
Tooth decay, mesial	0	1 (1.9 %)	
Twelve months follow-up			
Evaluation^‡^			0.001^†^
Code 00	42 (41.6 %)	27 (51.9 %)	
Code 10	7 (6.9 %)	11 (21.2 %)	
Code 11	1 (1.0 %)	0	
Code 30	14 (13.9 %)	4 (7.7 %)	
Child died	12 (11.9 %)	0	
Type of failure			
Partial loss, distal	3 (3.0 %)	7 (13.3 %)	
Partial loss, distal with repair need	1 (1.0 %)	0	
Partial loss, mesial	3 (3.0 %)	4 (7.7 %)	
Total loss	13 (12.9 %)	4 (7.7 %)	

**Fig. 2:**
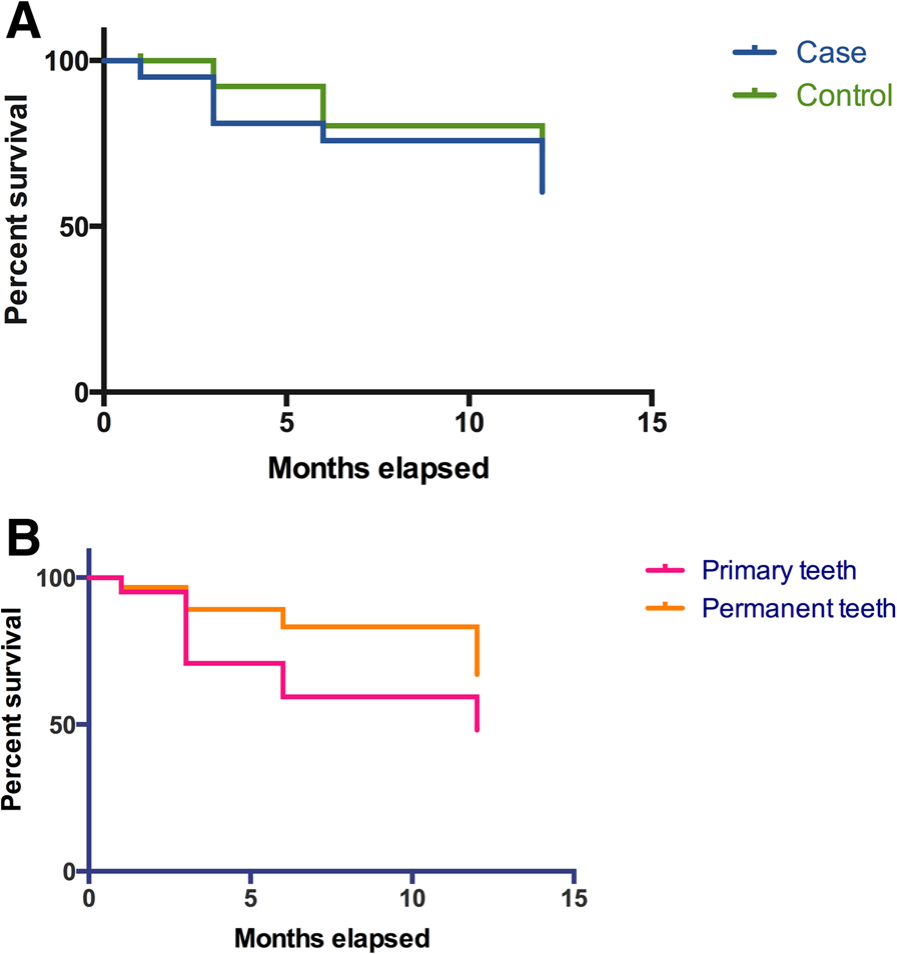
Life tables for the survival of ART comparing intervention groups (**a**) and dentition (**b**)

ART survival did not significantly differ among groups (putting together single surface restorations and sealants), but was different when comparing primary with permanent teeth, regardless of oncohematological treatment (Fig. 2 and Table 4). The survival life tables showed that at the twelfth month, 60.4 % of ART in the experimental group did not need repair compared with 72.6 % in the control group (*P* = 0.12), whilst 48.1 % of primary and 67.1 % of permanent teeth restored did not need repair (*P* = 0.01). ART in primary teeth had a 2.1× greater chance of failure or need for repair in a period of 12 months of follow-up, compared with permanent teeth (Table 4).

**Table 4:** Final result of Cox regression for occurrence of ART failure needing repair

Variables	Odds ratio (OR)	95 % confidence interval for OR	*P*
Group			
Experimental	1.6	0.8–2.9	0.155
Control	1		
Type of tooth			
Primary	2.1	1.2–3.7	0.008
Permanent	1		

As the majority of ART procedures were sealants, we ran a separate survival analysis excluding the ART single surface restorations (*n* = 23). The results showed that, at the 12-month assessment, 57.9 % of the ART sealants in the experimental group had survived without needing a repair, whilst in the control group the success survival rate was 78.0 % (*P* = 0.03).

Power estimation for the survival analyses varied from small to moderate, with an alpha of 0.05: 0.22 (ART restorations and sealants curve; experimental versus control group), 0.69 (ART comparing primary versus permanent teeth) and 0.65 (ART sealants only, experimental versus control group).

## Discussion

The main finding of this study was that survival rates of ART restorations and sealants placed in children undergoing oncohematological treatment were similar to those of healthy children, after 12 months, although the type of failure differed between groups. However, when ART sealants were analyzed separately, the experimental group had more failures. This partially confirms our hypothesis that ART in the experimental group would have a shorter survival. In addition, a secondary finding of this study was that ART longevity differed between primary and permanent teeth, confirming the literature’s findings as discussed in the next paragraphs.

The overall success rate of ART (codes 00 and 10), adding the experimental and the control group, varied from approximately 95–100 % in the first post-operative month and 48–73 % at the 12-month assessment. That is, there was a decreasein the success rate of almost 50 % in the experimental group over a 1-year period. Many studies have reported a relatively high longevity rate after 12 months of follow-up for single-surface ART restorations in primary teeth (94–99 %) [21–25] and in permanent ones (94–100 %) [26–32]. The differences in success rates among studies might be due to differences in the ART performance and evaluation criteria. Unfortunately, there are no reports in the literature on the success of other types of restorations in patients undergoing oncohematological treatment.

At the end of 12 months of follow-up, experimental and control groups did not differ regarding the need of repair according to the survival analysis (60.4 % versus 72.6 %, respectively), putting together ART restorations and sealants. However, this finding might be questioned if one considers that a difference of about 12 percentage points is clinically relevant. Maybe future studies with larger sample size are able to further test this difference. Interestingly, when the ART restoration cases were removed from the analysis, the interpretation of the results changed, because ART sealants survived longer in the control group. Given this fact, our team suggests future studies involving ART sealants and habits such as bruxism, for example.

The most observed failures were categorized as codes 11, 20 or 30 [19], which mean that most failures were related to mechanical issues with the restorations/sealants and not to secondary caries, which was observed in very few teeth. The experimental group had more failures in need of repair (code 30) and in the control group, most failure was related to slight repairs with no need for further repair (code 10).

Interestingly, we did not observe other codes of failure already reported in other studies, perhaps because there were relatively more pit and fissure sealants in our research.

Longer survival was expected in permanent teeth compared to primary teeth. A systematic review of the literature concluded that ART restorations and sealants in permanent teeth have fewer failures and less need for repair than restorations and sealants performed in primary teeth; i.e., restorations and sealants in permanent teeth present longer survival than those in deciduous teeth. This difference is likely due to increased adhesion between the glass ionomer cement and the enamel that occurs in permanent teeth, as they are more mineralized than the deciduous teeth [33]. This finding leads us to infer that young patients undergoing oncohematological treatment, i.e., those with primary molars should receive increased attention towards preventive procedures, as these teeth may be more prone to restoration failures than permanent teeth.

The ART restorations and sealants were performed and assessed by the same dentist. One of the advantages of the evaluation being conducted by only one surveyor was the accuracy of the technique. However, as the dentist was not blinded for the intervention group, there could be some bias in assessing the failures of restorations in the follow-up period.

Another important conclusion that can be extrapolated from our research is that although the ART technique presents longevity comparable to conventional restorative techniques, such as amalgam [34–38], in the group of patients undergoing oncohematological treatment, there were more failures in need of repair. Therefore, we can infer that it is extremely important that patients undergoing this type of treatment are regularly assessed by a dentist, who must repair any form of failure immediately after it is identified.

Our study had some limitations, including the small, non-calculated sample size, besides some expected limitations inherent to pragmatic trials [338]: the non-randomized allocation of interventions, the observer not masked to the procedure, the different settings where groups were submitted to intervention. Thus, the present results should be confirmed in larger trials. However, this research allows us to affirm that the ART technique is viable in the population investigated, as literature [14, 15, 33, 34] states that it provides benefits such as minimally invasive procedures, psychological comfort to the patient, the sealing of open cavities, and increased availability of fluoride in the oral cavity, among others.

Furthermore, it is important to note that this study is original and novel. Future multicenter studies with larger sample size and longer follow-up period should confirm our results, as well as investigate the quality of life and patients’ perceptions regarding atraumatic dental treatment. This may contribute to the recommendation of dental protocols for this population.

## Conclusions

Overall, this study shows that ART restorations and sealants in children undergoing oncohematological treatment are effective and feasible, but the occurrence of failures in need of repair indicates that the ART approach in this group of patients should be recommended as long as a systematic follow-up is planned and executed.

## Additional file

Additional file 1: Table S1. Criteria for follow-up evaluation of ART restorations and sealants (according to Gemert-Schriks, 2007). (DOCX 11 kb)

## Abbreviations

ABO-TO: Brazilian Dental Association – Tocantins Section
ALL: Acute lymphocytic leukemia
ART: Atraumatic restorative treatment
BFM-90: European group protocol Berlin-Frankfurt-Munster
C: Control
CI: Confidence interval
dmft: Index of decayed, missing and filled primary teeth
DMFT: Index of decayed, missing and filled permanent teeth
E: Experimental
OR: Odds ratio
REBEC: Brazilian registry of clinical trials
