# Supplementary material for: One-year follow-up of Atraumatic Restorative Treatment(ART) for dental caries in children undergoing oncohematological treatment: a pragmatic trial
Source: BMC Oral Health. 2015 Oct 16;15:127. doi: 10.1186/s12903-015-0110-y (PMC4609041; doi:10.1186/s12903-015-0110-y)
Supplement: Additional file 1: Table S1. — Criteria for follow-up evaluation of ART restorations and sealants (according to Gemert-Schriks, 2007). (DOCX 11 kb) [file 12903_2015_110_MOESM1_ESM.docx]

Additional file 1: Table S1. Criteria for follow-up evaluation of ART restorations and sealants (according to Gemert-Schriks, 2007)

| **Code** | **Evaluation characteristics** |
| --- | --- |
| 00 | Restoration present, correct |
| 10 | Restoration present, slight marginal defect/wear of surface (<0.5 mm). No repair needed. |
| 11 | Restoration present, gross marginal defect/wear of surface  (>0.5 mm). Repair needed. |
| 12 | Restoration present, underfilled (>0.5 mm). Repair needed. |
| 13 | Restoration present, overfilled (>0.5 mm). Repair needed. |
| 20 | Secondary caries, discoloration in depth, surface hard and  intact, caries within dentin. Repair needed. |
| 21 | Secondary caries, surface defect, caries within dentin. Repair  needed. |
| 30 | Restoration not present, bulk fracture, moving or partial lost. Repair needed. |
| 40 | Inflammation of the pulp; signs of dentogenic infection  (abscesses, fistulae, pain complaints). Restoration might still  be in situ. Extraction needed. |
| 50 | Tooth not present because of extraction |
| 60 | Tooth not present because of shedding |
| 70 | Tooth not present because of extraction or shedding |
| 90 | Patient not present |
